# Free-standing acute inpatient rehabilitation hospital enhanced practices and policies in response to the COVID-19 outbreak

**DOI:** 10.2144/fsoa-2020-0181

**Published:** 2020-12-09

**Authors:** Reza Ehsanian, Jessi Workman, Derrick Jones, David Selvage, W Evan Rivers, Athanasios K Manole, John Henry Sloan

**Affiliations:** 1 Division of Physical Medicine & Rehabilitation, Department of Department of Orthopedics & Rehabilitation, University of New Mexico School of Medicine, Albuquerque, NM, USA; 2 Lovelace Health System, Albuquerque, NM, USA; 3 New Mexico Department of Health, Healthcare-Associated Infections Program, Infectious Disease Epidemiology Bureau, Epidemiology & Response Division, Santa Fe, NM, USA; 4 Manzano Medical Group, Albuquerque, NM, USA

**Keywords:** acute rehabilitation, coronavirus disease, COVID-19, healthcare policy, severe acute respiratory syndrome coronavirus 2

## Abstract

This special report was developed to communicate policy and procedures for free-standing acute inpatient rehabilitation hospitals (AIRHs) to protect patients and healthcare personnel and to prevent further spread of severe acute respiratory syndrome coronavirus 2. The recommended policies were developed in conjunction with the New Mexico Department of Health and hospital leadership. As we attain additional knowledge and experience during this pandemic, suggestions of best practice will continue to evolve for AIRHs. The authors encourage readers to work with local regulatory officials to ensure regulatory compliance as well as respect of the availability of local resources.

## Lay abstract

This special report communicates potential policy and procedure modifications for free-standing acute inpatient rehabilitation hospitals in response to the severe acute respiratory syndrome coronavirus 2 (SARS-CoV-2)/coronavirus disease (COVID-19) pandemic. The goal of the communication is to help protect patients and healthcare personnel and to prevent further spread of SARS-CoV-2. The recommendations were developed in conjunction with the New Mexico Department of Health and hospital leadership. A comprehensive response policy to SARS-CoV-2/COVID-19 should include facility changes, enhanced personal protective equipment policy, enhanced admission policy and enhanced surveillance testing policy. In the setting of rapidly changing state and federal guidelines, hospital staff should work with state officials to ensure that potential policy and procedure modifications are in line with state and federal recommendations.

The severe acute respiratory syndrome coronavirus 2 (SARS-CoV-2), which is the infectious source for coronavirus disease (COVID-19) was first reported on 31 December 2019 [1]. As of 15 November 2020, there were 10,690,665 total positive cases of COVID-19 and 243,580 COVID-19 related deaths in the United States [2]. In New Mexico, there have been 63,171 total cases of individuals testing positive for COVID-19 out of 1,360,452 tests administered (4.6% positive) and 1208 COVID-related deaths [3]. There have also been 5523 total hospitalizations, with 480 being hospitalized as of 15 November 152020 [4]. In Bernalillo County, there have been 16,194 cases of individuals testing positive for COVID-19 (out of 439,038; 3.7% positive), with 256 deaths.

The rapid spread and sheer number of cases have exacted a global and national toll on the healthcare system as healthcare professionals balance the need for continued patient care with the need to control the spread of the virus [5]. This has been the case for all levels of patient care, including acute inpatient rehabilitation facilities, with societies such as the American Academy of Physical Medicine and Rehabilitation (AAPM&R) providing recommendations and resources to support care in the time of COVID-19 [6]. However, the authors agree with the AAPM&Rs statement that “Congress has been primarily focused on keeping COVID patients alive but hasn't moved on to considering what happens to patients after they survive. Many of these patients are still struggling with potentially long-term difficulties” [7]. More importantly, there has not been a consistent framework provided for free-standing acute inpatient rehabilitation facilities admitting patients with previous diagnosis of COVID-19, nor is there universally accepted guidelines for limiting the exposure of COVID-19 to the high-risk acute inpatient rehabilitation populations in these facilities.

Our free-standing acute inpatient rehabilitation facility is in a unique position because it is one of only four such facilities in the state of New Mexico capable of providing complex acute inpatient rehabilitation services. Our rehabilitation hospital is the only hospital in New Mexico accredited by the Commission on Accreditation of Rehabilitation Facilities for 3 years in six programs: Comprehensive Integrated Inpatient Rehabilitation Program, Inpatient Brain Injury Program, Inpatient and Outpatient Spinal Cord System of Care, Inpatient Stroke Specialty Program, Outpatient Medical Rehabilitation Program, and Outpatient Medical Rehabilitation Program (Children and Adolescents). The hospital has 265 employees with 43 contractors. There were 62 licensed acute inpatient rehabilitation beds and 1470 inpatient admissions in 2019. The hospital provides a full continuum of inpatient and outpatient rehabilitation care, including physical therapy, occupational therapy, speech and language pathology, rehabilitation nursing and case management services. The inpatient program is designed for patients with a variety of rehabilitation needs, offering general inpatient medical rehabilitation and three specialized programs: brain injury rehabilitation, stroke rehabilitation and spinal cord injury rehabilitation. Details of the program are supplied as Supplementary material to this article and can be found online [8].

On 6 July 2020, senior hospital leadership was informed of a patient who discharged on 3 July 2020 then tested positive for COVID-19 two days after being discharged. In response to this notification, all patients and staff were tested that same week as part of a swift and organized response. Testing resulted in identification of three patients and four staff members with positive COVID-19 tests. Given the acute inpatient rehabilitation population at our facility (e.g., older adults often with underlying chronic medical conditions) is at high risk of being affected by respiratory pathogens such as COVID-19, a comprehensive assessment was made with resulting action plans (Table 1).

**Table 1.** SARS-CoV-2/COVID-19 action plan and desired outcomes.

Action plan	Desired outcome
All hospital filters to be changed to MERV-13	Prevent or reduce the airborne transfer of infectious organisms and viruses through airborne transmission
Enhanced PPE Policy	Enhanced PPE (i.e., KN95, face shields) delivered and deployed
SARS-CoV-2/COVID-19 Enhanced Admission Policy	Prevent COVID-19–positive patient admission
SARS-CoV-2/COVID-19 Enhanced Surveillance Testing Policy	Identify staff and patient COVID-19 exposure and asymptomatic active infections
SARS-CoV-2/COVID-19 Updated Facility Policy	Maintain average census
Response to Positive SARS-CoV-2/COVID-19 Test	Limit likelihood of an undiagnosed COVID-19 exposure for staff and patients

MERV-13: Minimum efficiency reporting value-13; PPE: Personal protective equipment; SARS-CoV-2/COVID-19: Severe acute respiratory syndrome coronavirus 2/coronavirus disease.

Our hospital has an established infection prevention and control program. It became clear, however, that new measures were needed in the setting of this unique pandemic. The medical director, in conjunction with state officials from the Department of Health and senior hospital leadership, developed new procedures and policies as detailed below.

## Goals & specific aims of proposed SARS-CoV-2/COVID-19 enhanced policy to limit airborne transfer, enhanced admission, enhanced surveillance & updated facility policy

The goal of the SARS-CoV-2/COVID-19 specific policies and procedures is to protect patients and healthcare personnel and to prevent further spread of the virus. These policies and procedures may be of benefit to other free-standing acute inpatient rehabilitation facilities.

The specific aims of the program are as follows:

- Identify SARS-CoV-2 exposures
- Contain SARS-CoV-2 exposures
- Safely maintain average daily census

The efficient and timely identification of SARS-CoV-2 exposures mitigates risk of an outbreak across our hospital system and protects the vulnerable patient population. Containment limits exposures to a single unit, allowing for continued patient flow in other units, ultimately allowing for uninterrupted admission and the ability to maintain the average census. This enables ongoing, high-level rehabilitation services while also allowing admission from overwhelmed acute care hospitals.

## SARS-CoV-2/COVID-19 enhanced policy to limit airborne transfer

- To help prevent or reduce airborne transfer of infectious organisms and viruses through airborne transmission, all hospital filters were changed to minimum efficiency reporting value-13 (MERV-13). The MERV-13 filters are reported to filter 90% of particles in the 3–10 μm range, 85% of particles in the 1–3 μm range and 50% of particles in the range 0.3–1 μm range (exact ranges differ by brand, and the reader is encouraged to work with local officials to determine the most appropriate choice).
- Enhance personal protective equipment (PPE) (i.e., KN95 and face shields) were delivered and deployed to hospital staff. This enhanced PPE protocol was made mandatory; the use of cloth, homemade masks was initially discouraged and later banned.

## SARS-CoV-2/COVID-19 enhanced admission policy

### Revised patient admission policy

- All potential patients must be free of symptoms typically associated with SARS-CoV-2 for 7 days before admission, unless attributed to other etiologies (e.g., negative for COVID-19 and positive for influenza)
- All potential patients not previously identified as COVID-19 positive are required to have at least one negative test within 72 h before admission
- Patients previously identified as COVID-19 positive require two negative tests >24 h apart before admission

## SARS-CoV-2/COVID-19 enhanced surveillance testing policy

### Patient and staff testing

- All newly admitted patients are tested upon admission regardless of the last COVID-19 test
- All admitted patients tested weekly
- Weekly rotating surveillance testing of 25% hospital staff

## SARS-CoV-2/COVID-19 updated facility policy

**Figure 1.**
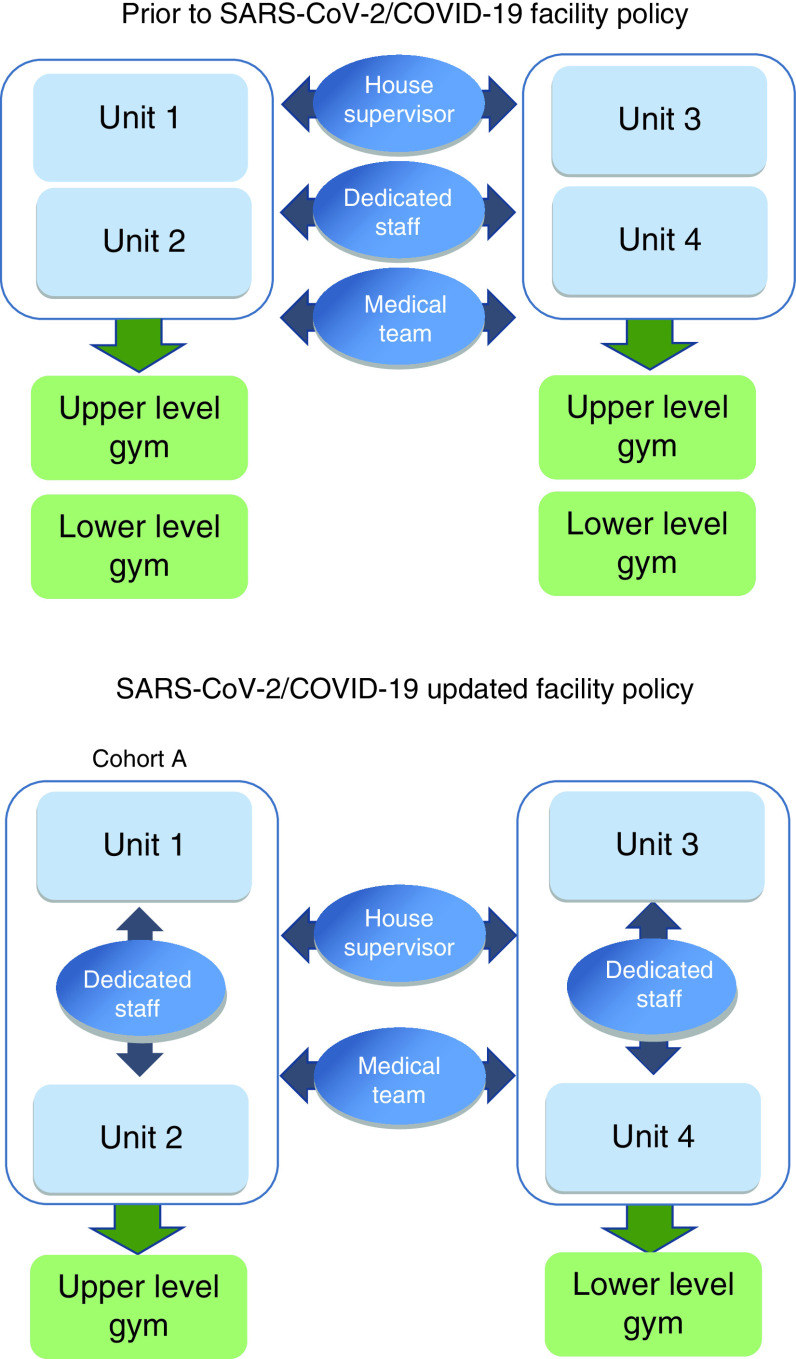
SARS-CoV-2/COVID-19 updated facility policy. Dedicated staff: nursing, therapy and ancillary staff. Medical team: medical doctors, nurse practitioners and physician assistants. SARS-CoV-2/COVID-19: Severe acute respiratory syndrome coronavirus 2/coronavirus disease.

- Cohorts established with designated units, rehabilitation gym space, nursing, therapy and ancillary staff (Figure 1)
- Each cohort consisting of 31 patient rooms (double occupancy) with dedicated nursing, therapy (assigned to unit) and ancillary staff (assigned to cohort)
- Nursing, therapy and ancillary staff cannot visit/aid units not in designated cohort
- Social distancing procedures [9] are enforced throughout facility including nursing stations, break rooms, work rooms and gyms

## Response to positive SARS-CoV-2/COVID-19 test

- Patients identified as positive through the admission testing or weekly surveillance testing process will be immediately transferred to acute care hospital for medical triage and treatment
- Affected unit would be quarantined for at least 2 weeks:

— Comingling between adjacent units in cohort will cease immediately and continue to be prohibited until such time as the quarantine is lifted

 — No new patients admitted to affected unit and no patients will be transferred out of affected unit other than to discharge (acute, home, or other appropriate facility)

- Designated staff responsible for cohort in which patient was assigned will undergo immediate testing and subsequent weekly testing until no new positive results are detected

## Discussion

The goal of the developed policy and procedures is to protect patients and healthcare personnel and to prevent further spread of SARS-CoV-2. The policies were developed in conjunction with an infectious disease epidemiologist at the New Mexico Department of Health. The authors feel that these processes and procedures may be of benefit to other free-standing acute inpatient rehabilitation facilities.

These suggestions for updated facility policies in response to the SARS-CoV-2/COVID-19 pandemic are in line with the experience of other facilities [10–15]. As we attain additional knowledge and experience during this pandemic, suggestions of best practice will continue to evolve for acute inpatient rehabilitation hospitals. However, as we develop policies and procedures, prior practice guidelines, such as those learned from severe acute respiratory syndrome should be considered [15].

The development of our hospital's policies and procedures follows current recommendations and prior experience with respiratory pandemics. In the setting of rapidly changing state and federal guidelines we also worked with New Mexico state officials to ensure our recommendations were in line with state and federal recommendations. The authors encourage readers to work with local regulatory officials to ensure any polices or procedures developed are within regulatory compliance, respects the availability of local resources and addresses specific local conditions.

## Future perspective

There will be a need to develop detailed federal and national guidelines for acute inpatient rehabilitation hospitals in response to SARS-CoV-2/COVID-19 pandemic and future viral pandemics. These guidelines will ensure rehabilitation hospital policies and procedures are within regulatory compliance, respect the availability of national and local resources and appropriately address the evolving pandemic. In the absence of such guidelines, rehabilitation hospitals will have to work with state officials to develop guidelines in response to the SARS-CoV-2/COVID-19 pandemic.

**Executive summary**

- Policy and procedures should be developed for acute inpatient rehabilitation hospitals to protect patients and healthcare personnel and to prevent further spread of severe acute respiratory syndrome coronavirus 2 (SARS-CoV-2)/coronavirus disease (COVID-19).
- In the setting of rapidly changing state and federal guidelines hospital staff should work with state officials to ensure recommendations are in line with state and federal recommendations.
- A comprehensive response policy to SARS-CoV-2/COVID-19 should include facility changes, enhanced personal protective equipment policy, enhanced admission policy and enhanced surveillance testing policy.
